# Occupational pesticide exposure increases risk of acute myeloid leukemia: a meta-analysis of case–control studies including 3,955 cases and 9,948 controls

**DOI:** 10.1038/s41598-021-81604-x

**Published:** 2021-01-21

**Authors:** Amelie Foucault, Nicolas Vallet, Noemie Ravalet, Frederic Picou, Marie C. Bene, Emmanuel Gyan, Olivier Herault

**Affiliations:** 1 Department of Biological Hematology, Tours University Hospital, Tours, France; 2 CNRS ERL7001 LNOX, EA 7501, Tours University, Tours, France; 3 Department of Hematology and Cell Therapy, Tours University Hospital, Tours, France; 4 Department of Biological Hematology, Nantes University Hospital, Nantes, France

**Keywords:** Acute myeloid leukaemia, Risk factors, Acute myeloid leukaemia, Occupational health

## Abstract

The impact of pesticides on health is a major public health concern. A higher risk to develop chronic lymphoid malignancies has been demonstrated to be associated with occupational pesticide exposure (OPE). By contrast, little is known of the impact of OPE on the occurrence of myeloid malignancies especially acute myeloid leukemia (AML). The purpose of this meta-analysis is to summarize data on the association between OPE and AML. A relevant dataset of case–control studies was extracted. Among 6784 references extracted, 14 were selected, representing 3,955 AML patients and 9,948 control subjects diagnosed between 1976 and 2010. An adverse association was found between OPE and AML (OR = 1.51; 95%CI: 1.10–2.08), not affected by sensitivity analyses. Funnel plot asymmetry suggested a publication bias underestimating OR. Stratified analysis showed the association to be driven by studies with: (1) monocentric AML patients and hospital-based control population, (2) Newcastle–Ottawa scale > 6 and the group of studies identified as with the lowest risk, (3) exposure assessment through peer-to-peer interview, (4) diagnosis in North America and Asia and after 1995, (5) restriction to de novo AML. Moreover, the association between OPE and AML was significant with insecticides. These findings broaden the spectrum of pesticide toxicity to myeloid malignancies.

## Introduction

The worldwide usage of pesticides has been steadily increasing since the 90′s, from 1.5 kg/ha in 1990 to 2.6 kg/ha in 2016^1^. Thus, potential deleterious effects on human health of pesticide exposure are a current major public health concern. Pesticides include a large variety of molecules, classified according to their target (herbicides, fungicides, insecticides)^2^.

Lymphoid and myeloid neoplasms are classified by the World Health Organization (WHO) according to clinical, histological, cytological, immunophenotypic and molecular characteristics^3,4^. Molecular mechanisms leading to chronic and acute hematological malignancies are heterogeneous and impact different types of hematopoietic precursor cells^3,4^. The carcinogenic effects of pesticides have been thoroughly investigated in lymphoid hematological malignancies, revealing a pejorative impact of occupational pesticide exposure (OPE) on the incidence and outcome of non-Hodgkin lymphoma^5–11^. This association has not been clearly demonstrated to date in myeloid malignancies, especially acute myeloid leukemia (AML). This is due to the inappropriate definition of “AML”, often pooled with other leukemias (lymphoid, acute and chronic myeloid leukemia) and poorly characterized^12–14^. Moreover, because pathogenesis varies from one hematological malignancy to another, the impact of OPE cannot be generalized^3,4^.

AML include several subtypes of myeloid malignancies based on cytological, immunophenotypic, cytogenetic or molecular characteristics^3,15^. AML is the most common type of acute leukemia in adults, accounting for about 1% of all cancers^16^. In myelodysplastic syndromes (MDS), known to be pre-leukemic diseases^3,15^, a meta-analysis of case–control studies revealed an association with OPE (odds ratio [OR] = 1.95; 95%CI: 1.23–3.09)^17^. Regarding myeloid leukemias, a meta-analysis performed in 2007 included 17 cohort and 16 case–control studies focused on chronic and acute myeloid leukemia. In the AML subgroup, this study reported an association between OPE and AML exclusively in cohort studies (relative risk [RR]: 1.55; 95%CI: 1.02–2.34) but not in case–control studies^18^. This difference is possibly due to the case–control studies method that exposes to recall bias^19^. Additionally, the risk of AML in this meta-analysis was possibly underestimated since cancer patients, who have a higher risk of developing AML^20^, were included as controls in three (25%) studies. Finally, in three other studies, AML cases were exclusively identified based on the cause of death on death certificates, well known to be imprecise^21^ and only selecting non-surviving patients obviously unable to answer questionnaires.

It thus appeared to us that a new meta-analysis of case–control studies, including more recent publications and based on stringent inclusion criteria, was necessary to possibly reinforce the results of cohort studies and confirm that OPE increases the risk of AML. Following the Preferred Reporting Items of Systematic reviews and Meta-Analyses (PRISMA) statement^22^, this meta-analysis of 14 studies published between 1986 and 2017 confirmed a significant adverse association between OPE and AML (OR = 1.51; 95%CI: 1.10–2.08) which further supports the impact of pesticides on AML pathogenesis.

## Methods

### Literature search strategy and selection process

According to PRISMA statement^22^, an extensive search was performed to identify manuscripts reporting studies that evaluated an association between adult AML and OPE. The MEDLINE, EMBASE and Cochrane databases were exploited from 1946 to 2020. References and abstracts were extracted on September 1st, 2020. To identify studies reporting any type of pesticides molecule and knowing that pesticides may be referenced as different types of “chemicals”^2^ or appear in demographic studies, three search strategies were used, focused on pesticides, demographics and chemical exposure data. These strategies are described in Supplementary Methods. Firstly, references were hand-selected according to title and abstracts and classified as relevant or irrelevant. Secondly, eligibility was assessed through full text review and articles classified according to their inclusion or main reason for non-inclusion. Each step of the systematic review process was performed concomitantly and independently by two investigators of this study (AF and NV). Disagreements were resolved by consensus and when no consensus was achieved, the opinion of two other investigators (OH and EG) was asked for. Results of the first and second steps are detailed in Supplementary Datasets 1 and 2.

### Eligibility

Stringent inclusion criteria were chosen to limit the risk of bias regarding AML incidence and pesticide exposure. Studies meeting the following criteria were included: (1) case–control design, (2) available data specific for AML patients (excluding pooled leukemias), (3) AML diagnosis excluding patients identified only through death certificates, (4) controls not restricted to cancer databases, (5) specific report of exposure to pesticides, including all studies with any kind of pesticide exposure, one class of pesticide exposure or indirect chronic exposure during work or any activity (6) sufficient data to analyze (number of patients *AND*/*OR* odd ratio *AND*/*OR* adjusted odd ratio with 95%CI), (7) adult patients (≥ 15 years-old), (8) English language. In case of overlapping study populations, the most recent and detailed study was chosen for the meta-analysis. Unpublished studies and abstracts were not considered.

### Data extraction

Extracted data are described in Supplementary Methods. No author was contacted for further information. Data extraction was concomitantly and independently performed by two investigators of this study (AF and NV). Disagreements were resolved by consensus or two investigators (OH and EG).

### Quality assessment

Quality assessment of the study was conducted using the well-described Newcastle–Ottawa scale (NOS)^23^. This scale describes each case–control study according to selection, comparability and exposure definition as well as assessment criteria. Because lack of reproducibility is criticized with NOS^24^, none of the studies was excluded on the basis of this score and, as mentioned above, two investigators (AF and NV) independently assessed each study and confronted their results. Disagreements were resolved by consensus or by the opinion of two other investigators (OH and EG).

### Bias and confounding factors

Known AML risk factors are age, male gender, exposure to ionizing agents or benzene, previous cytotoxic treatment, MDS or genetic predispositions^25^. It turned out to be impossible to adjust our meta-analysis estimate for these known confounding factors, due to the lack of individual participant level data. However, possible confounding factors were controlled with (1) inclusion criteria described above and (2) use of adjusted OR.

It was next planned to describe possible bias within and across studies according to Grading of Recommendations, Assessment, Development and Evaluation (GRADE) Working Group guidelines^26^. A three levels scale was used to describe remaining bias such as source populations of cases and controls, exposure assessment, confounding factors and funding. These three levels of bias risk, respectively low, unclear and high, refer to plausible bias that are respectively unlikely, likely and seriously associated with weakening of the final results (levels are defined in Table S1). Again, bias risks were evaluated independently (AF and NV) and disagreements resolved by consensus or external review (OH and EG). Correspondence analysis was used to describe the distribution of levels of risk of bias between studies^27^.

### Data synthesis and statistical analyses

Studies characteristics are reported with descriptive statistics. Considering the differences of studied populations and methods of exposure assessment that yielded heterogeneity, the DerSimonian and Laird random effects model was used^28^. Data were summarized by the number of individuals exposed or not to pesticides, with or without AML. The association was quantified using OR with a 95% confidence interval. Adjusted OR and 95%CI from each study were preferred. If unreported, unadjusted OR and 95%CI with the reported number of exposed and non-exposed cases and controls were calculated. For the final analysis, OR and standard error of OR were used. Cochrane’s Review Manager calculator was used for OR and standard error calculations^29^. Statistical analyses were performed by NV and FP with the “metafor” package^28^ of the R software (v3.4.0)^30^.

### Sensitivity analysis

To evaluate the stability of the results, a sensitivity analysis was conducted by sequential omission of each study and inclusion of alternative OR analyses when present. A time-dependent cumulative analysis was performed.

### Publication bias

Publication bias were assessed graphically with funnel plots to highlight asymmetry and with Egger’s test^31^. A trim-and-fill analysis was performed in case of suspected asymmetry in order to correct the OR using R_0_, L_0_ and Q_0_ estimators^32^. This method imputes hypothetical unpublished studies to mirror those published and calculates an adjusted OR to assess the impact of these hypothetical missing studies on the pooled estimate.

### Subgroup analysis

To identify which main sources of factors influenced the final OR, subgroup analyses was performed. Subgroups were defined from study design (AML population, control population, NOS score, source of bias), pesticide exposure recording methods (exposure assessment, job exposure matrix or expert review), confounding factors analyses (matched, adjusted OR), studied population (geographical zone, AML type, time period) and pesticide subtypes as reported in included studies.

## Results

### Literature search and eligibility

**Figure 1:**
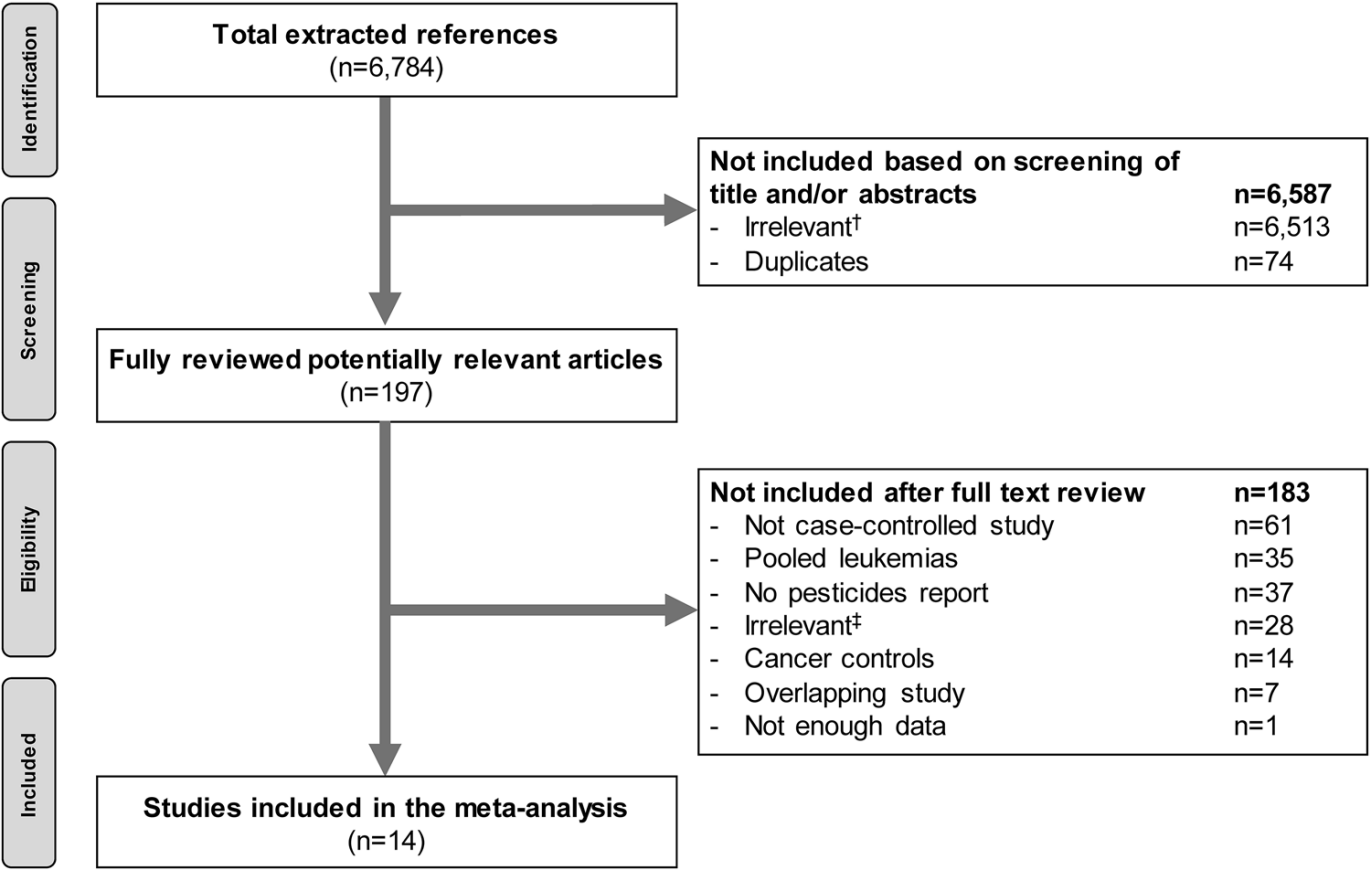
Flow diagram of study selection. ^†^in vitro*/*in vivo studies, case reports, non-English publications, cohort description, non-acute myeloid leukemia (AML) patients, pooled analysis of different leukemia subtypes. ^‡^in vitro studies, reviews, case report, erratum, conference paper, death as outcome, missing data on the outcome and exposure, non-AML patients. References are available in Supplementary Datasets 1 and 2.

The search strategy identified 6,784 references. After abstracts assessment, 197 references were retained. Full text review finally excluded 183 references. Therefore, 14 studies were included in the meta-analysis (Fig. 1)^33–46^.

### Study characteristics

The main characteristics of the 14 studies are reported on Table 1. Inclusion periods were heterogeneous as illustrated in Fig S1. The median duration of inclusions was 4 years (range: 1–17).

**Table 1:** Main characteristics of case–control studies included in the meta-analysis evaluating the association of AML and pesticide exposure.

First author	Year	Country	Inclusion periods	Gender	Inclusion age	AML source^a^	Controls source^a^	Matching criteria	Adjusted OR^b^	No. AML	No. controls	Exposure^c^	Quality score (/9)^d^
Flodin U^33^	1986	Sweden	1977–1982	M/F	20–70	Multicentric	Population	Age, gender, residency	No	59	354	SAQ	4
Richardson S^34^	1992	France	1984–1988	M/F	> 30	Multicentric	Hospital	Age, gender, ethnicity, residency	Yes	154	513	PTP	8
Crane MM^35^	1992	USA	1982–1983	M/F	> 18	Multicentric	Hospital	Age, gender, ethnicity, year of inclusion	No	60	60	PTP	7
Ciccone G^36^	1993	Italy	1989–1990	M/F	15–74	Monocentric	Hospital and population	Age, gender	Yes	50	246	PTP	6
Mele A^37^	1994	Italy	1986–1990	M/F	> 15	Multicentric	Hospital	NS	Yes	252	1,161	PTP	4
Albin M^38^	2000	Sweden	1976–1993	M/F	> 20	Multicentric	Population	Age, gender, residency	No	372	351	PTP	6
Lazarov D^39^	2000	UK, Yugoslavia	1986–1994	M/F	> 18	Multicentric	Hospital	Age, gender, year of inclusion	No	98	196	PTP	7
Adegoke OJ^40^	2003	China	1987–1989	M/F	> 15	Multicentric	Population	Age, gender	Yes	236	502	PTP	6
Terry PD^41^	2005	USA, Canada	1986–1989	M/F	17–79	Multicentric	Population	Age, gender, ethnicity, residency	Yes	599	607	PTP	6
Kaufman DW^42^	2009	Thailand	1997–2003	M/F	> 18	Monocentric	Hospital	Age, gender, residency	Yes	87	756	PTP	7
Wong O^43^	2010	China	2003–2007	M/F	> 18	Multicentric	Hospital	Age, gender, hospital	No	722	1,444	PTP	8
Strom SS^44^	2012	USA	2003–2007	M/F	18–80	Monocentric	Population	Age, gender, ethnicity, residency	No	638	636	PTP	8
Parodi S^45^	2017	Italy	1990–1993	M/F	20–74	Multicentric	Population	Age, gender	No	223	1,774	PTP	7
Poynter JN^46^	2017	USA	2005–2010	M/F	20–79	Multicentric	Population	Age	Yes	405	1,348	SAQ	6

^a^Acute myeloid leukemia (AML) patients and controls source are described in Table S3; ^b^Adjustment variables are fully described in Table S6; ^c^Pesticide exposure methods are fully described in Table S2; ^d^Quality score was evaluated with the Newcastle–Ottawa scale for case–control studies (Table S4). NS: not specified; PTP: person-to-person interview; SAQ: self-administered questionnaire; OR: odds ratio.

The meta-analysis ultimately included 3,955 AML patients and 9,948 controls. The main source of AML patients was multicentric (n = 11). Five studies reported only de novo AML. One reported 0.69% of secondary AML (sAML). The other studies (n = 8) did not report whether the AML were de novo, sAML or therapy-related (tAML). Age at inclusion was heterogeneous but most patients were between 40 and 75 years-old (Fig S2). Control cohorts were hospital-based (n = 5 studies), general population-based (n = 8) or both (n = 1). AML patients and controls were matched in all but one study. Reported matching criteria were age (n = 13 studies), gender (n = 11), residency (n = 5), ethnicity (n = 4), year of inclusion (n = 2) and hospital (n = 1) (Table 1).

Exposure was evaluated through self-administered questionnaires in two studies, while twelve used peer-to-peer interviews (Table 1 and Table S2). In addition, six studies evaluated the probability of OPE with expert reviews or job matrix exposure (Table S2). Seven studies were conducted in Europe, four in North America and three in Asia. The median study quality NOS score was 6.5 (range: 4–8) (Table S4). No direct conflict of interest with pesticide industries was disclosed (Table S5).

The total numbers of exposed subjects were at least 563 AML patients and 1,219 controls (excluding one study which did not report these numbers^38^). Three studies reported alternative OR according to the duration of OPE^35,40,44^. Seven studies that reported adjusted OR and adjusted covariates are depicted in Table S6^34,36,37,40–42,46^. Only four studies specified OR for the types of pesticides (herbicides n = 4; insecticides n = 3) and two considered the modality of application (fumigants n = 2)^34,37,43,46^. Only one study reported the results of exposure for specific molecules in cases and controls^42^.

### Bias study

**Figure 2:**
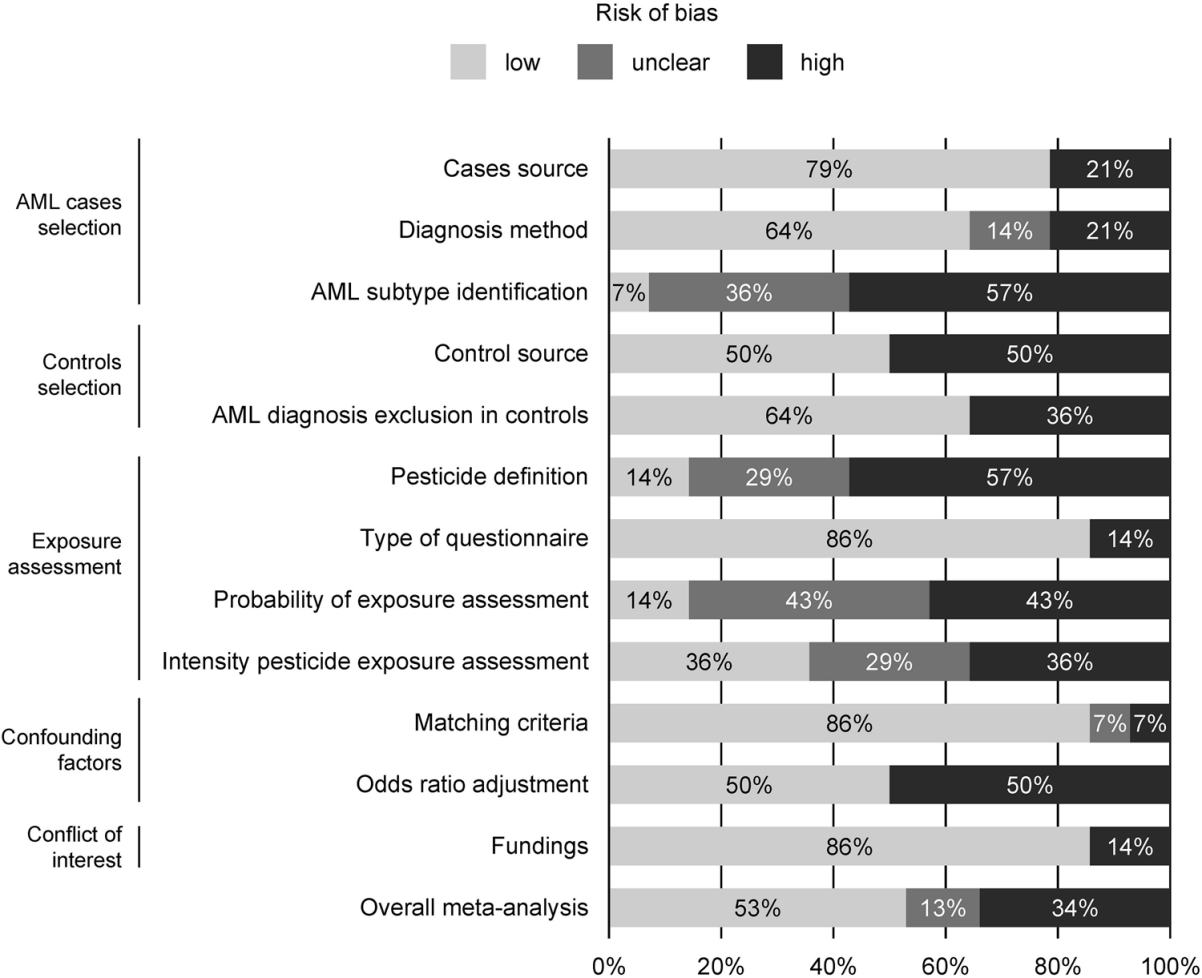
Risk of bias across studies assessed following Grading of Recommendations, Assessment, Development and Evaluation (GRADE) Working Group guidelines. Low, unclear and high, refer to plausible bias that are respectively unlikely, likely and seriously associated with weakening of the final results (defined in Table S1). Results in individual studies are reported in Fig S3.

Plausible bias in the meta-analysis and within each study are summarized in Fig. 2 and Fig S3A. Bias were found in AML subtype diagnosis according to cytogenetic or molecular abnormalities. Pesticides definition was classified as unclear or high source of bias in 29% and 57% of studies. The intensity of pesticide exposure was also poorly described.

In correspondence analysis, dimension 1 was driven by unclear and high risk of bias (76% and 24%) while low risk of bias contributed to 48% of dimension 2. Four studies were associated with a higher rate of high risk^33,35,37,45^. Using the distribution of studies in the second dimension, six studies were identified as associated with lower risk of bias^34,40,42–44,46^ (Fig S3B).

### Risk estimation

**Figure 3:**
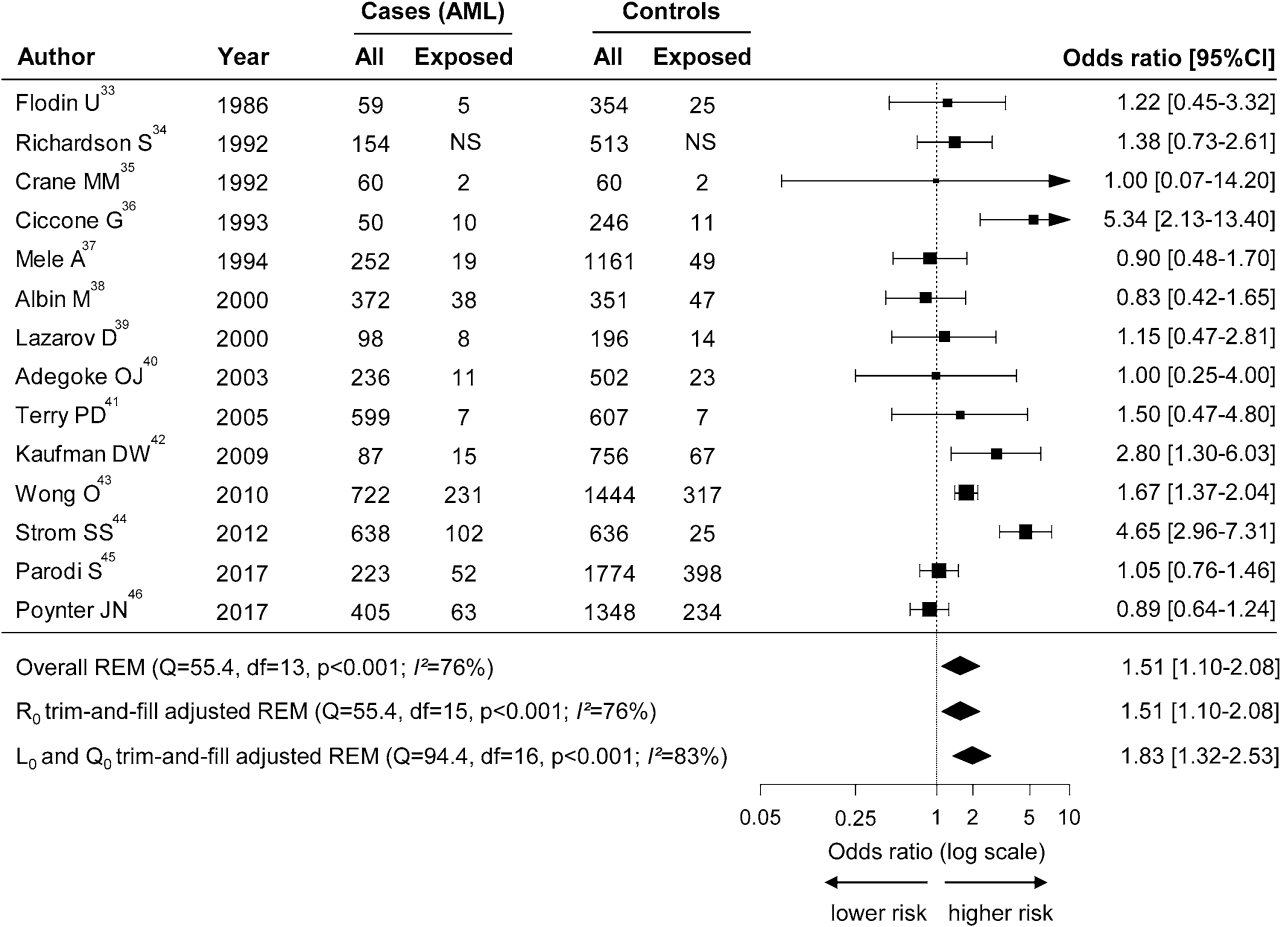
Forest plot illustrating the overall significant increase of acute myeloid leukemia (AML) risk in populations exposed to occupational pesticides. The overall cumulative analysis was calculated with adjusted odds ratio (OR) whenever available, using the random effect model (REM). Pesticide exposure had to be clearly stated in each study, its definition being reported in Table S2. Matching and adjustments strategies are reported in Table 1 and Table S6. Squares indicate the OR for each study, solid diamonds show the pooled meta-analysis estimates and error bars are defined as the 95% confidence interval (95%CI). The size of squares correlates to the weight of each study in the meta-analysis. Publication bias adjustments using trim-and-fill methodology are reported below the overall REM. Imputed analyses are shown in Fig. 5. Q: Cochrane Q-test, df: degree of freedom; p: *p* value of the heterogeneity test; NS: not specified; superscript numbers correspond to the list of references. This figure was performed using R software^30^ with the “metafor” package^28^.

Using a random effects model, the overall analysis showed a significant adverse association between OPE and AML with OR = 1.51 (95%CI: 1.10–2.08) (Fig. 3), and a significant heterogeneity between studies (*I*^2^ = 76%, *p* < 0.001).

### Sensitivity analyses and time-dependent meta-analysis

**Figure 4:**
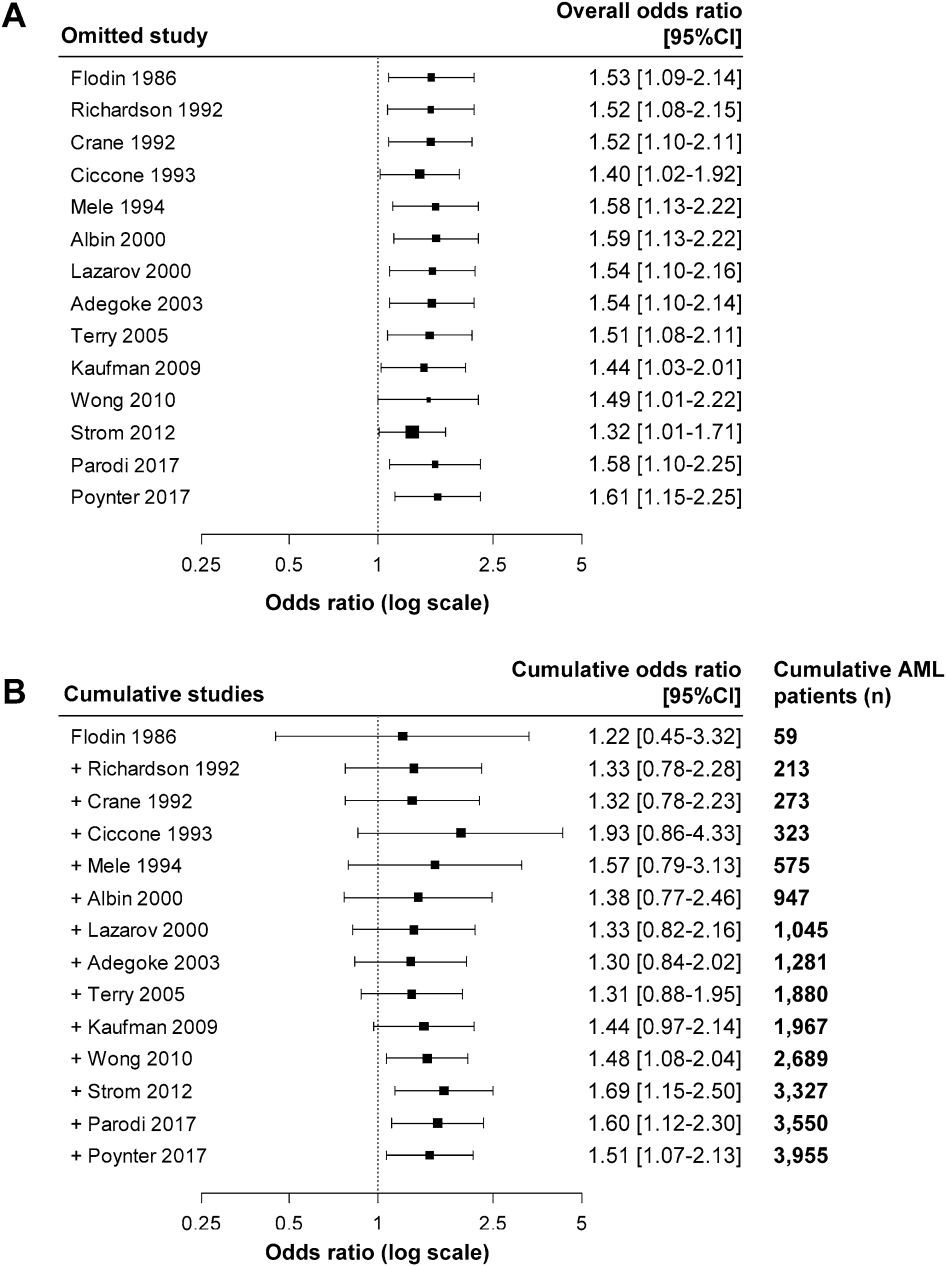
Sensitivity and time-dependent cumulative analyses. (**A**) Sensitivity analysis by sequential omission of every study. Each line refers to the global odds ratio (OR) after excluding the corresponding study. In all cases, the OR is statistically significantly in favor of a risk of developing acute myeloid leukemia (AML) related to occupational pesticide exposure (OPE). (**B**) Time-dependent analyses after adding each study down to the full 14 publications. The OR, in favor of AML risk related to OPE, becomes statistically significant after the cumulative inclusion of 2,689 patients (from Wong O et al.). This figure was performed using R software^30^ with the “metafor” package^28^.

None of the studies was identified as outlier by Q–Q plot (Fig S4). Sequential one-by-one exclusion of the studies did not influence the overall estimate. OR ranged from 1.32 (95%CI: 1.01–1.71) to 1.61 (95%CI: 1.15–2.25) (Fig. 4A). When the studies were added chronologically by publication date, the 95%CI narrowed progressively and became significant from 2010 on (Fig. 4B). Using alternative lower OR, the overall estimate remained statistically significant (OR = 1.40 [95%CI: 1.06–1.86]).

One screened but not included study reported individual OR according to plasmatic levels of organochlorines-derived-metabolites. It was not eligible for inclusion in this meta-analysis because of lack of individual data to calculate the overall OR (Fig. 1)^47^. Nevertheless, due to the objective biological assessment of pesticide exposure used in this publication, we calculated the impact of individual OR on the results of our meta-analysis. OR remained statistically significant, ranging from 1.44 (95%CI: 1.05–1.98) to 1.51 (95%CI: 1.10–2.08) (Table S6 and S7).

### Publication bias

**Figure 5:**
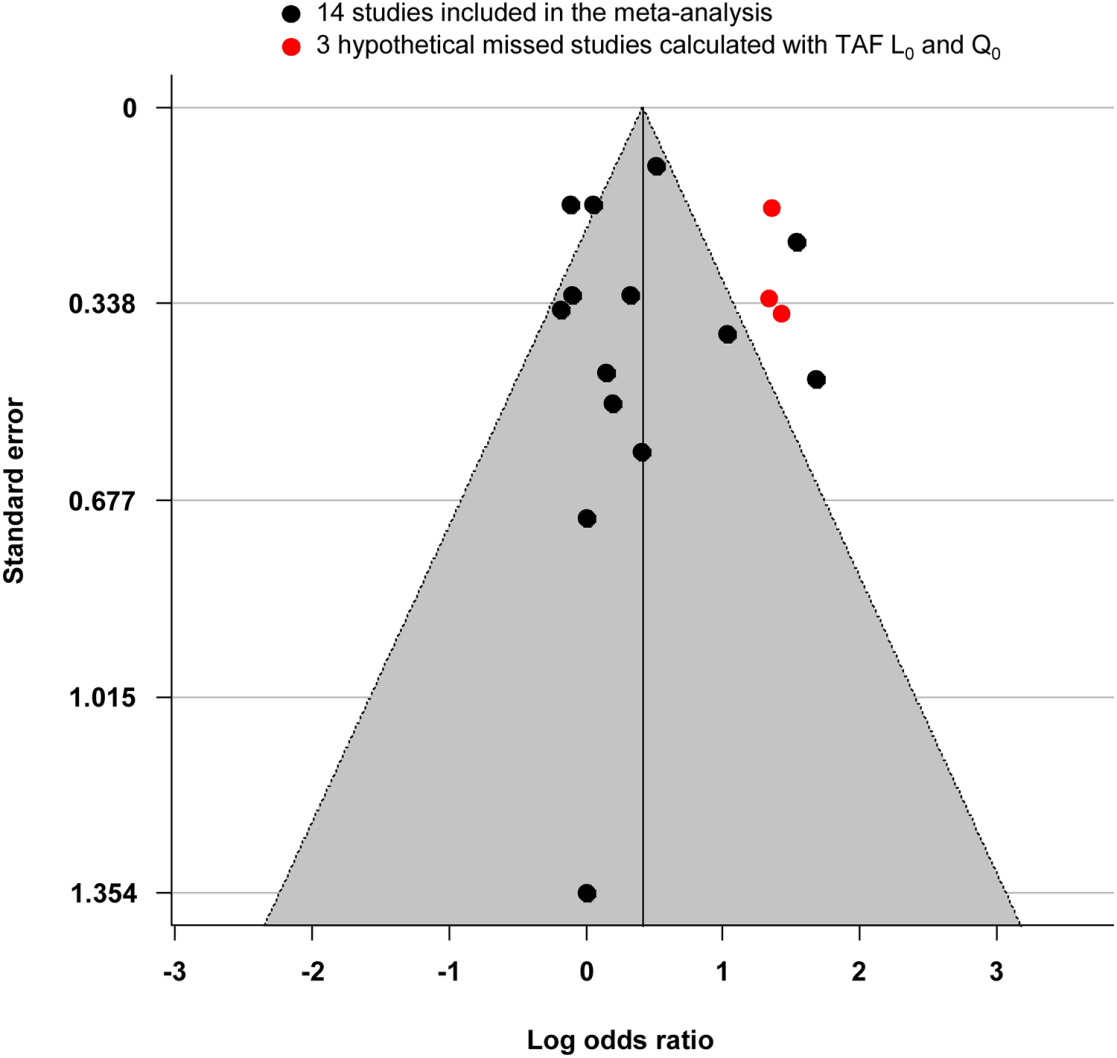
Funnel plot illustrating publication bias analysis. Logarithmic risk estimates are plotted against the standard error for each study. The plot is centered around the overall effect with 95% confidence region estimated effect based on the random effect model. Black circles denote identified studies and their summary measures. Red circles represent missing studies and their hypothetical measures after adjustment for funnel plot asymmetry using trim-and-fill (TAF) L_0_ and Q_0_ estimators, respectively. R_0_ did not identify missing studies. This figure was performed using R software^30^ with the “metafor” package^28^.

Even though the Egger’s test was not statistically significant (*p* = 0.95), graphical analysis of the funnel plot revealed an asymmetry that suggested a publication bias (Fig. 5). Using L_0_ and Q_0_ estimators, trim-and-fill analysis imputed studies in order to correct the OR (OR = 1.83 [95%CI: 1.32–2.53]). The R_0_ estimator did not identify the studies that could be imputed (Figs. 1, 5).

### Stratified analysis

**Figure 6:**
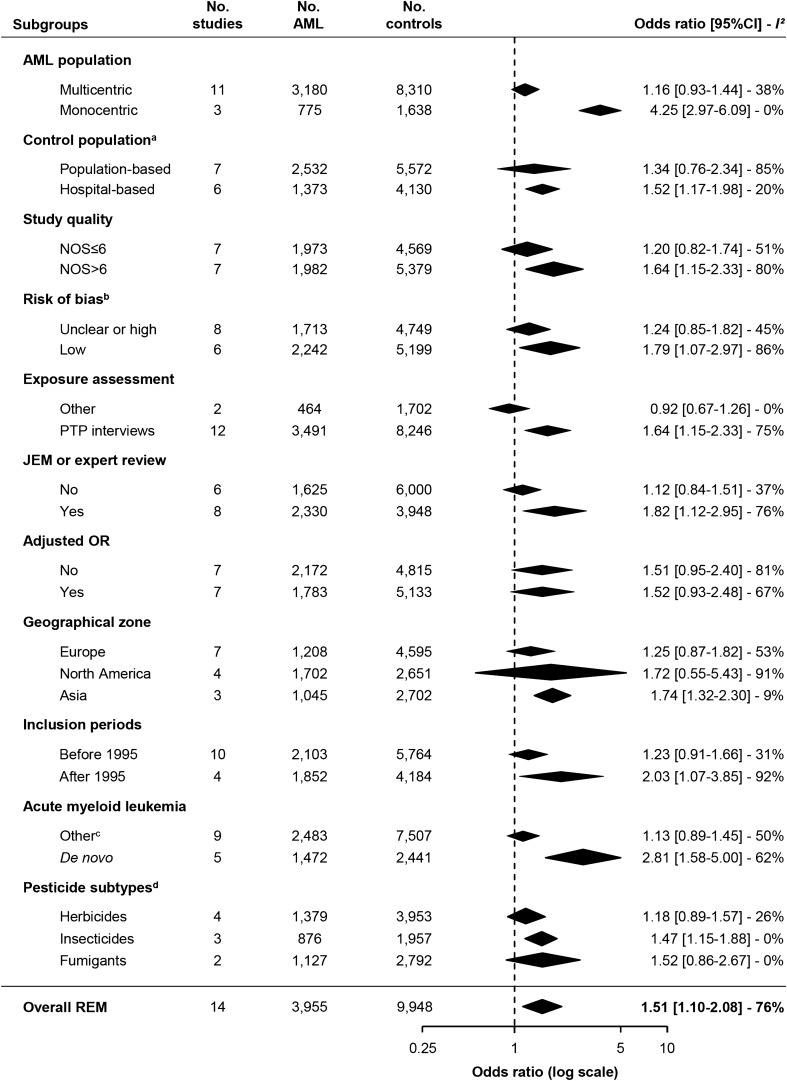
Forest plot illustrating the risk estimates and their 95% confidence intervals (95%CI) within subgroups of included studies. Subgroups are presented in ascending order according to their mean effects. The center and width of diamonds represent mean OR and 95%CI. Heterogeneity within each stratus is reported with *I*^2^. ^a^Cicconne et al.^36^ not included: hospital- and population-based; ^b^risk of bias classification was performed using correspondence analysis described in Fig S3; ^c^other: de novo status was not mentioned or 0.69% of secondary acute myeloid leukemia (sAML) in Wong et al.^43^; ^d ^only studies reporting specific pesticide exposure were included for this subset analysis. NOS: Newcastle–Ottawa scale; PTP: peer-to-peer interviews. Description of articles and number of subjects included in each stratus are shown in Table S8. This figure was performed using R software^30^ with the “metafor” package^28^.

All studies were stratified to explore factors influencing overall OR (Fig. 6 and Table S8). This stratified analysis showed that the association between OPE and AML was notably stronger in studies with (1) monocentric AML patients and hospital-bases control population (2) NOS > 6 and the group of studies identified as those with lowest risk, (3) exposure assessment through peer-to-peer interview, (4) diagnosis in North America and Asia and after 1995, (5) restriction to de novo AML. Regarding the type of pesticide, when known, the association between OPE and AML was significant with insecticides (OR = 1.45 [95%CI: 1.16–1.81]). Strata of adjusted and non-adjusted OR studies were similar.

### Quality of evidence

According to GRADE approach, quality of evidence (QoE) reflects the confidence that an estimate effect is close to the observed effect^26^. QoE can be categorized in four grades: very low (very little confidence), low (limited confidence), moderate (moderately confidence), high (very confident). The GRADE approach to rating the QoE begins with the study design. Since GRADE guidelines^26^ were designed for interventional studies, it must be adapted to observational studies, such as human health impacts of environmental exposure. In this context, the level of evidence of case–control studies can be defined as moderate, just after prospective cohort studies which present the highest level of evidence. The next step was to study the factors modulating QoE. Heterogeneity (*I*^2^ = 76%), indirectness of evidence (due to the use of standardized questionnaires), imprecision and bias across studies could decrease the certainty of evidence to a lower level. This is counterbalanced by higher OR in studies with lower risk of bias or with higher quality score, by publication bias underestimating the OR, and by the large effect calculated. Consequently, the overall certainty of evidence of this meta-analysis can be considered as moderate (Table 2).

**Table 2:** GRADE summary of findings table.

Outcome	No. participants (studies)	Overall inclusion periods	Geographical zone	Quality of evidence	OR (95%CI)
AML diagnosis	13,903 (14)	1976 to 2010	Europe (Sweden, France, Italy, United Kingdom, Yugoslavia) North America (USA, Canada) Asia (China, Thailand)	⨁⨁⨁**◯** Moderate	1.51 (1.10–2.08)

## Discussion

Knowledge about the impact of OPE on cancer risk is a major societal concern. There are increased evidences of the association of hematological lymphoid malignancies and OPE, but little is known regarding AML. This meta-analysis of highly selected case–control studies similarly demonstrates an adverse association between OPE and the risk of developing AML.

Observational cohort studies prospectively collect exposure data conversely to case–control studies in which data are retrospectively collected. Because of this, recall bias is inherent to the design of case–control studies^48^, notably in case of occupational exposure, which requires the participant to recall the details of many years of employment. Nevertheless, when considering occupational risk, individuals usually have a good memory of the jobs carried out during their lifetime, even if they are not able to remember the details and specifications of all the pesticides they used. Moreover, the risk of recall bias is reduced with the use of standardized questionnaires, expert assessment and job exposure matrices. The possible financial benefits, or social factors, associated with the recognition of this risk can potentially lead to either an over- or under-estimation of exposure^48^.

The meta-analysis performed in 2007 by van Maele-Fabry et al. reported an association between OPE and AML in cohort studies but not in case–control studies^18^. This may be partly explained by recall bias and also by the inclusion of controls with past history of cancer, which increases the risk of AML (from 1.2-fold for colon cancer up to 19.3-fold for bones and joints malignancies)^20^. Moreover, identification of patients through death certificates could induce bias due to errors in reporting the diagnosis of AML^21^. Our aim was to avoid these biases by only selecting studies without these characteristics.

Expecting to include all studies reporting OPE and AML diagnosis, it was decided to use a broad strategy in order to identify all references published since 1946 containing information about AML incidence and OPE. The keywords used were “pesticides”, “demographics” and “chemicals”. This allowed for the identification of four references not disclosed with only the “pesticides” keyword^35,38,39,44^. The references identified were compared with those from the 2007 meta-analysis^18^.

This method ultimately selected 14 studies of interest from 6,784 literature references. These stringent criteria allowed the inclusion of six studies^33,34,36,37,40,41^ from the previous work^18^. Moreover, three unused references published before 2007 were added^34,35,43^. Finally, 5 new studies published since 2007^42–46^ were included.

Using a random effects model, the overall OR estimate of the meta-analysis was 1.51 (95%CI: 1.10–2.08), pooling adjusted OR (when reported) and unadjusted OR. Interestingly, this result is similar to the OR calculated from cohort studies (OR = 1.55)^17^. All studies but one were matched for confounding factors^40^. Seven studies reported adjusted OR for pesticide exposure^34–37,40–42,46^. Adjusted ORs were added to the final model to limit the risk of bias with potential confounding factors. However, in subgroup analysis, there were no differences in OR between adjusted and unadjusted studies.

The quantitative graphical heterogeneity identified in the Forest plot was confirmed by the heterogeneity test (I^2^ = 76%). This could be explained by (i) different geographical zones studied, (ii) heterogeneous inclusion periods (ranging from 1976 to 2010), (iii) study design (matching criteria, adjustment covariates, source of AML patients and controls), (iv) patient characteristics, notably age ranging from 15 to over 75 years old and v) OPE assessment methods.

The stability of the overall OR was checked by (1) sequential exclusion, which did not show major influence from any single study, (2) an alternative model with lower exposure intensities reported ORs which did not influence the overall OR. Moreover, adding individual OR of pesticides-derived metabolites as recently reported by Bassig et al.^47^ did not influence the results.

In time-dependent cumulative analyses, it appeared that OR became significant from 2010 on, when the study by Wong et al.^43^ was added, possibly explaining the lack of association between AML and OPE in case–control studies reported by van Maele-Fabry et al.^18^ which appeared before this publication. However, this should not be interpreted as an increase in AML risk with increased pesticide use over the years, considering that the date of publication does not reflect the period of inclusion. Indeed, one of the five reports published after 2009 included patients from the period 1990–2000^45^.

No publication bias was found using Egger’s test. However, this test is low-powered in moderate bias identification^31^ and graphical analysis indeed contrarily suggested a publication bias with a higher number of references on the left side of the funnel plot. A trim-and-fill method was used to explore the potential effects of missed studies that impacted funnel plot symmetry^32^. Two estimators calculated an overall bias-corrected OR by adding dummy studies so that funnel plot symmetry was reached. Furthermore, studies reporting pesticides exposure as subgroups not identified in the title, abstract or keywords might have been missed with our strategy.

The overall quality of selected studies was illustrated and compared in a standardized way by using NOS^23^. Twelve studies ranked between 6 and 8, suggesting that most of the included references followed recommendations on the report of case–control studies. In subgroup analyses, these highly ranked references showed a higher OR (1.80 vs. 1.20). Even if this scale suffers from potential variability, it was under control with the independent reviewing of each study by two of the authors, followed by consensus^24^. Knowing the limits of NOS, we elected to include all studies although NOS scores varied from 4 to 8. The impact of study quality based on NOS was then evaluated in the stratified analysis which confirmed that the subgroup with a higher NOS also had a higher OR (1.80 vs. 1.20). NOS is a generic tool for assessing the overall methodology quality. We thus described three levels of plausible bias respectively from the cases, controls and exposure assessment and in confounding factors within each. Six studies were identified that were more likely to be associated with a lower risk of bias and for which the OR was higher than in the eight others (2.03 vs. 1.03)^34,40,42–44,46^. These results suggest that the association between OPE and AML observed in this meta-analysis is partly driven by studies with lower bias and higher quality.

The types of AML (de novo, sAML or tAML) were also extracted during the full review process. They were reported in six studies, five having included only de novo AML^36,39,41,42,44^, and the last one 99% and 1% of de novo and sAML, respectively^43^. The association with OPE appeared to be stronger in de novo AML studies than in others (OR = 2.81 vs. OR = 1.13, respectively). In order to estimate the frequency of sAML in all included references, the age at inclusion was extracted. Most patients were between 40 and 75 years of age. Five studies included patients over 75 years-old (representing a minority of the population included)^37,40–42,46^ while three studies included some patients below the age of 18 (minimum 15 years-old)^37,40,41^. It is established that the median age at transformation ranges between 62 and 64 years old in MDS-sAML and non-MDS-sAML^49^. Thus, we can postulate that the populations studied may have included sAML or tAML; even if not specified. Previous MDS in included patients was not mentioned in the references retained. Since MDS may evolve to AML, the association between AML and OPE probably also takes into account the OPE-induced risk of MDS^17^.

Extracted data on AML types are further limited since data concerning OPE and molecular findings in AML were not reported. Older studies compared cytogenetic abnormalities in exposed and non-exposed patients but none found any significant impact of an abnormal karyotype nor abnormalities of chromosomes 5 or 7^35,36,50^. However, these studies described small cohorts.

The source of patients and controls may be a cause of bias to properly estimate the risk of OPE-related AML. AML patients were mainly from multicentric studies (n = 11 studies), while control sources were hospital- and population-based (n = 5 and n = 8, respectively). The comorbidities of control patients from hospitals may influence AML incidence. Moreover, cities and geographical zones may impact on the access to hospital (notably university hospitals) and consultation rate. By considering geographical zone subgroups, the highest association between OPE and AML risk was observed in Asia, followed by North America and Europe. This result could be explained by a higher usage of pesticides in Asia and the USA, compared to European countries^1^. Likewise, the population included after 1995 was associated with a significantly higher OR (2.03 vs. 1.23) which may reflect the increase on pesticide usage since the 90′s^1^.

OPE assessment was evaluated through self-administered questionnaires or peer-to-peer interviews. Seven studies controlled self-reported assessments by expert review (n = 5)^34–36,38,39^ or job-exposure matrices (n = 2)^40,44^ which may improve the accuracy of exposure classification. In subgroup analysis, the association between OPE and AML was stronger using publications reporting peer-to-peer interviews (1.64 vs. 0.92) and matrix or expert consensus (1.82 vs. 1.12). As suggested by the bias analysis, pesticide definition was poorly described in the studies included. The WHO defines pesticides as “chemical compound(s) used to kill pests including insects, rodents, fungi and unwanted plants”^51^. This definition refers to the function of these products and is insufficient regarding the vast number of molecules, their mechanisms of action and their application methods^2^. Additionally, adjuvants in pesticides are generally declared to be inert, but have been indicated to amplify the toxicity of the active ingredient^52^. Four studies specified pesticides as their different categories (herbicides, insecticides, etc.) while others did not provide any precise definition of pesticides^34,37,43,46^. Only one study reported the details on these molecules^42^. The way in which pesticides are used is also a factor to consider in the risk of AML, since subgroup analysis using the mode of pesticides exposure revealed a stronger association between OPE to fumigants and AML (OR = 1.52). The type of pesticides is also probably important to consider, since it has been reported that insecticide exposure is associated with an increased risk of MDS (OR = 1.71; 95%CI: 1.22–2.40)^17^.

High and unclear overall risks of bias were estimated at 40% and 14% following the GRADE method, respectively^26^. Because the GRADE approach is well-suited for clinical studies which evaluate pharmacological or technical treatment intervention but not environmental studies, we adapted GRADE criteria to the context of this meta-analysis in order to objectively assess the risk of bias^53^. When considering QoE assessment, the same approach was used. The factors decreasing QoE, such as heterogeneity and indirectness were counterbalanced by those increasing it such as higher OR in studies with lower risk of bias. Following this approach, the quality of evidence was estimated as moderate: “the true effect is likely to be close to the estimate of the effect, but there is a possibility that it is substantially different” ^26^.

Even if an association between OPE and AML is demonstrated by this meta-analysis, the causal interpretation should be formulated with caution. The selected studies reported OR with uncontrolled confounding factors between controls and AML patients, since AML characteristics and risk factors are poorly reported in studies investigating the relationship between AML and OPE^3,15,25^, as already observed in the previous meta-analysis^18^. Likewise, dose–response links are critical information to discuss causality. Here, exposure doses were only reported in three references^35,40,44^. Among these, one observed a higher risk of AML in the highly exposed group^44^. In stratified analysis, OR was higher in North American and Asian population as well as in inclusion periods after 1995. This result may also support a dose-relation effect considering that pesticides usage is higher in these populations (Fig S5)^2^.

## Conclusions

In lymphoid malignancies, a higher rate of non-Hodgkin’s lymphomas (NHL) was first described among farmers, then cohort and case–control studies confirmed and described the specific association between pesticides molecules families and an increased risk of NHL^5,6,8^. To date, such data on specific molecules have not been reported in AML. By performing a meta-analysis with highly selected case–control studies, we report here on a documented adverse association between OPE and AML risk. This result reinforces the results of cohort studies published 12 years ago and is a new argument to consider AML as an occupational illness in patients with demonstrated OPE. As for lymphoid diseases, further studies will have to focus on the biological effects of individual pesticides and agricultural cocktails, in order to determine how these molecules are involved in leukemogenesis.

## Supplementary Information

Supplementary Information 1.

Supplementary Information 2.

Supplementary Information 3.

Supplementary Information 4.

## Data Availability

All published data collected during the systematic review are available in this publication and its supplementary information files.

## References

[1] FAO (Food & Agriculture Organization), http://www.fao.org/faostat/en/#data/EP/visualize (Accessed: April 4, 2018).

[2] Pesticide Action Network, North America, PAN Pesticide Database, http://www.pesticideinfo.org (Accessed: April 4, 2019).

[3] Arber DA (2016). The 2016 revision to the World Health Organization classification of myeloid neoplasms and acute leukemia. Blood.

[4] Swerdlow SH (2016). The 2016 revision of the World Health Organization classification of lymphoid neoplasms. Blood.

[5] Blair A, Zahm SH (1995). Agricultural exposures and cancer. Environ. Health Perspect..

[6] De Roos AJ (2003). Integrative assessment of multiple pesticides as risk factors for non-Hodgkin’s lymphoma among men. Occup. Environ. Med..

[7] Fritschi L (2005). Occupational exposure to pesticides and risk of Non-Hodgkin’s lymphoma. Am. J. Epidemiol..

[8] Leon ME (2019). Pesticide use and risk of non-Hodgkin lymphoid malignancies in agricultural cohorts from France, Norway and the USA: a pooled analysis from the AGRICOH consortium. Int. J. Epidemiol..

[9] Lynch, S.M., Mahajan, R., Beane Freeman, L.E., Hoppin, J.A. & Alavanja, M.C. Cancer incidence among pesticide applicators exposed to butylate in the Agricultural Health Study AHS. *Environ. Res* **109**, 860–868 (2009). 10.1016/j.envres.2009.06.006 PMC3799990 19615679

[10] Zheng T, Zahm SH, Cantor KP, Weisenburger DD, Zhang Y, Blair A (2001). Agricultural exposure to carbamate pesticides and risk of non-Hodgkin lymphoma. J. Occup. Environ. Med..

[11] Lamure S (2019). Association of occupational pesticide exposure with immunochemotherapy response and survival among patients with diffuse large B-cell lymphoma. JAMA New Open.

[12] Viel JF, Richardson S (1991). Adult leukemia and farm pratices: an alternative approach for assessing geographical pesticide exposure. Soc. Sci. Med..

[13] Sperati A, Rapiti E, Settimi L, Quercia A, Terenzoni B, Forastiere F (1999). Mortality among male licensed pesticide users and their wives. Am. J. Ind. Med..

[14] Kristensen P, Andersen A, Irgens LM, Laake P, Bye AS (1996). Incidence and risk factors of cancer among men and women in Norwegian agriculture. Scand. J. Work Environ. Health.

[15] Döhner H (2017). Diagnosis and management of AML in adults: 2017 ELN recommendations from an international expert panel. Blood.

[16] American Cancer Society.https://www.cancer.org/cancer/acute-myeloid-leukemia/about/key-statistics.html (Accessed: April 4, 2018).

[17] Jin J (2014). Pesticide exposure as a risk factor for myelodysplastic syndromes: A meta-analysis based on 1,942 cases and 5,359 controls. PLoS ONE.

[18] Van Maele-Fabry G, Duhayon S, Lison D (2007). A systematic review of myeloid leukemias and occupational pesticide exposure. Cancer Causes Control.

[19] Schulz KF, Grimes DA (2002). Case-control studies: research in reverse. Lancet.

[20] Morton LM (2013). Evolving risk of therapy-related acute myeloid leukemia following cancer chemotherapy among adults in the United States, 1975–2008. Blood.

[21] Johansson LA, Westerling R (2000). Comparing Swedish hospital discharge records with death certificates: implications for mortality statistics. Int. J. Epidemiol..

[22] Moher, D., Liberati, A., Tetzlaff, J., & Altman, D.G., PRISMA Group. Preferred reporting items for systematic reviews and meta-analyses The PRISMA statement. *PLoS Med.* **62**, 1006-10012 (2009). PMC3090117 21603045

[23] Wells, G.A. *et al.* The Newcastle-Ottawa Scale (NOS) for assessing the quality if nonrandomized studies in meta-analyses, http://www.ohri.ca/programs/clinical_epidemiology/oxford.asp (Accessed: August 2, 2018).

[24] Stang A (2010). Critical evaluation of the Newcastle–Ottawa scale for the assessment of the quality of nonrandomized studies in meta-analyses. Eur. J. Epidemiol..

[25] Deschler B, Lübbert M (2006). Acute myeloid leukemia: Epidemiology and etiology. Cancer.

[26] Schünemann, H., Brożek, J., Guyatt, G. & Oxman, A. *GRADE Handbook, Cochrane Training*. http://www.training.cochrane.org/resource/grade-handbook. (Accessed: January 2, 2019).

[27] Lê, S., Josse, J. & Husson, F. FactoMineR : An R Package for Multivariate Analysis. *J Stat Softw.* **25**, 1–18. http://www.jstatsoft.org/v25/i01. (Accessed: January 2, 2019) (2008).

[28] Viechtbauer W (2010). Conducting meta-analyses in R with the metafor package. J. Stat. Softw..

[29] Review Manager (RevMan) [Computer program]. Version 5.3. Copenhagen: The Nordic Cochrane Centre, The Cochrane Collaboration (2014).

[30] R Core Team. R: A Language and environment for statistical computing. http://www.R-project.org (2017).

[31] Sterne JAC, Gavaghan D, Egger M (2000). Publication and related bias in meta-analysis: Power of statistical tests and prevalence in the literature. J Clin. Epidemiol..

[32] Shi L, Lin L (2019). The trim-and- fill method for publication bias: practical guidelines and recommendations based on a large database of meta-analyses. Medicine.

[33] Flodin U, Fredriksson M, Persson B, Hardell L, Axelson O (1986). Background radiation, electrical work, and some other exposures associated with acute myeloid leukemia in a case-referent study. Arch. Environ. Health.

[34] Richardson S (1992). Occupational risk factors for acute leukaemia: a case-control study. Int. J. Epidemiol..

[35] Crane MM, Annegers JF, Godwin JE, Keating MJ (1992). Is histological subtype a marker for environmental exposures in acute myelogenous leukemia?. Cancer Epidemiol. Biomark. Prev..

[36] Ciccone G (1993). Myeloid leukemias and myelodysplastic syndromes: Chemical exposure, histologic subtype and cytogenetics in a case-control study. Cancer Genet. Cytogenet..

[37] Mele A (1994). Hair dye use and other risk factors for leukemia and pre-leukemia: a case-control study. Italian Leukemia Study Group. Am. J. Epidemiol..

[38] Albin M (2000). Acute myeloid leukemia and clonal chromosome aberrations in relation to past exposure to organic solvents. Scand. J. Work Environ. Health.

[39] Lazarov D, Waldron HA, Pejin D (2000). Acute myeloid leukaemia and exposure to organic solvents–a case-control study. Eur. J. Epidemiol..

[40] Adegoke OJ (2003). Occupational history and exposure and the risk of adult leukemia in Shanghai. Ann. Epidemiol..

[41] Terry PD, Shore DL, Rauscher GH, Sandler DP (2005). Occupation, hobbies, and acute leukemia in adults. Leuk Res..

[42] Kaufman DW, Anderson TE, Issaragrisil S (2009). Risk factors for leukemia in Thailand. Ann. Hematol..

[43] Wong O, Harris F, Armstrong TW, Hua F (2010). A hospital-based case-control study of acute myeloid leukemia in Shanghai: Analysis of environmental and occupational risk factors by subtypes of the WHO classification. Chem. Biol. Interact..

[44] Strom SS, Oum R, Elhor Gbito KY, Garcia-Manero G, Yamamura Y (2012). De novo acute myeloid leukemia risk factors. Cancer.

[45] Parodi S (2017). Coffee and tea consumption and risk of leukaemia in an adult population: A reanalysis of the Italian multicentre case-control study. Cancer Epidemiol..

[46] Poynter JN (2017). Chemical exposures and risk of acute myeloid leukemia and myelodysplastic syndromes in a population-based study. Int. J. Cancer.

[47] Bassig BA (2019). Pre-diagnostic serum concentrations of organochlorines and risk of acute myeloid leukemia: A nested case-control study in the Norwegian Janus Serum Bank Cohort. Environ. Int..

[48] Coughlin SS (1990). Recall bias in epidemiologic studies. J. Clin. Epidemiol..

[49] Granfeldt Østgård LS (2015). Epidemiology and clinical significance of secondary and therapy-related acute myeloid leukemia: A national population-based cohort study. J. Clin. Oncol..

[50] Cuneo A (1992). Morphologic, immunologic and cytogenetic studies in acute myeloid leukemia following occupational exposure to pesticides and organic solvents. Leuk. Res..

[51] World Health Organization. http://www.who.int/topics/pesticides/en. (Accessed: April 2, 2018).

[52] Mesnage, R., Defarge, N., Spiroux de Vendômois, J., Séralini, G.E. Major pesticides are more toxic to human cells than their declared active principles. *Biomed Res Int.* **2014**, 179691 (2014). 10.1155/2014/179691 PMC3955666 24719846

[53] Morgan, L. R., *et al.* GRADE: Assessing the quality of evidence in environmental and occupational health. *Environ. Int.* **92–93**, 611–616 (2016). 10.1016/j.envint.2016.01.004 PMC4902742 26827182

