# Supplementary material for: Occupational pesticide exposure increases risk of acute myeloid leukemia: a meta-analysis of case–control studies including 3,955 cases and 9,948 controls
Source: Sci Rep. 2021 Jan 21;11:2007. doi: 10.1038/s41598-021-81604-x (PMC7820275; doi:10.1038/s41598-021-81604-x)
Supplement: Supplementary file 4 — Supplementary Information 4. [file 41598_2021_81604_MOESM4_ESM.pdf]

Occupational pesticide exposure increases risk of acute myeloid leukemia: a meta-analysis of case-control studies including 3,955 cases and 9,948 controls

Amelie Foucault, Nicolas Vallet, Noemie Ravalet, Frédéric Picou, Marie C Bene, Emmanuel Gyan, and Olivier Herault

SUPPLEMENTARY METHODS

Literature search strategy

Extracted data

SUPPLEMENTARY TABLES AND FIGURES

Table S1. Classification of risk of bias used in this meta-analysis.

Table S2. Details on exposure assessment in included studies.

Table S3. Details on acute myeloid leukemia (AML) patients and control sources.

Table S4. Detailed Newcastle-Ottawa scale evaluation for studies included in the meta-analysis.

Table S5. Reported funding of included studies.

Table S6. Variables included in the final model from the seven studies reporting adjusted odds ratios.

Table S7. Sensitivity analysis including results from the study of Bassig *et al.*

Table S8. Stratified pooled odds ratio of the relationship between pesticide exposure and risk of acute myeloid leukemia.

Fig S1. Inclusion periods and date of publication of included studies in the meta-analysis.

Fig S2. Graphical representation of inclusion age heterogeneity between included studies.

Fig S3. Bias assessment within each study.

Fig S4. Outlier studies identification with Q-Q plot.

Fig S5. Frequency of acute myeloid leukemia over time together with pesticide usage in the USA.

SUPPLEMENTARY METHODS

Literature search strategy

The following search strategies were used: (i) "(acute myeloid leukemia OR myeloid leukemia OR AML OR acute leukaemia) AND (pesticide OR herbicide OR rodenticide OR insecticide OR fungicide OR repellent OR pest)"; (ii) "(acute myeloid leukemia OR myeloid leukemia OR AML OR acute leukaemia) AND (chemicals OR solvents OR organochlorine OR alachlor OR propachlor OR halogenated hydrocarbon OR benzene)"; (iii) "(acute myeloid leukemia OR myeloid leukemia OR AML OR acute leukaemia) AND (occupation OR farmers OR agriculture OR rural)". To focus on adult populations, for every search we added "NOT (child OR childhood OR children OR infant)". The spelling "leukaemia" was also used to increase the number of screened references. No language restrictions were placed within the search strategy but only English-language publications were ultimately retained. The references extracted were compared to those of the previously published meta-analysis[18].

Extracted data

The following data were extracted: study quality score (see below), inclusion period, age of included AML patients, country of origin, source of AML patients, source of controls, number of total and exposed AML patients and controls, matching covariates, unadjusted OR, adjusted OR, covariates of adjustment, methods for exposure assessment, pesticides definition, reported funding. Alternative OR for different exposure intensities were also extracted. These items were used for the stratified analysis.

Table S1. Classification of risk of bias used in this meta-analysis.

		Low	Unclear	High
**Cases (AML)**	**Cases source**	Multicentric	-	Monocentric
	**AML method**	WHO or FAB criteria	reviewed by experts	ICD, other or not specified
	**AML subtype identification**	AML subtype defined with cytogenetic or molecular findings	AML only defined as de novo, secondary or therapy related	other or not specified
**Control (non AML)**	**Source**	population	-	hospital
	**AML diagnosis exclusion in controls**	exclusion AML diagnosis	-	no information on AML diagnosis exclusion
**Exposure assessment**	**Pesticide definition**	clear definition with identified molecules	at least one usage or type of pesticides stated in the manuscript (insecticides, rodenticides, etc.)	pesticide definition not specified
	**Type of questionnaire**	peer to peer interviews	-	other or not specified
	**Probability of exposure evaluation**	use of job exposure matrix	expert review	other or not specified
	**Intensity of pesticide exposure**	described	not clear	not specified
**Cofounding factors**	**Matching**	matched gender and age	matched gender or age	other or not specified
	**Adjustment**	adjusted studies	-	no adjustment or not specified
**Funding**		no direct relation with pesticides industry	-	relationship with industries or funding not specified

WHO: world health organization; FAB: French-American-British; ICD: international classification of diseases; AML: acute myeloid leukemia

Table S2. Details on exposure assessment in included studies.

First author and reference	Keywords used for the report or assessment of OPE	Additional tool to evaluate the probability of exposure	Source of exposure definition	Metrics of exposure measurement	Lookback time prior to diagnosis	Participation fractions for cases	Participation fractions for controls
Flodin, U.[33]	Pesticides (not otherwise specified)	NS	Mailed questionnaire	NS	NS	100%	90.8%
Richardson, S.[34]	Pesticides, insecticides, weed killers	Expert review	Detailed questionnaire following a standardized interview and assessed by a professional	high (>50% of the working time), medium (5-50%) exposure and low (<5%)	NS	72%	95%
Crane, M.M.[35]	Pesticides (not otherwise specified)	Expert review	Interview by one of the first two authors using a questionnaire	NS	NS	98%	100%
Ciccone, G.[36]	Pesticides (not otherwise specified)	4 levels probability by expert review	Questionnaire collected broad range of possible risk factors in addition to solvent exposure. The descriptions of the participants' tasks were then categorized in terms of their potential exposure	1) not exposed; 2) exposed to some of the agents listed, but not to the one under investigation; 3) "possibly" exposed (low probability of exposure) ; 4) "probably" exposed	NS	91%	99% hospital<br>82% population
Mele, A.[37]	Pesticides, herbicides	NS	Interviews following a questionnaire pilot-tested in a small sample	Several occupations described: agricultural workers, professional use of herbicides, professional use of pesticides, greenhouse workers	NS	99.6%	100%
Albin, M.[38]	Pesticides (not otherwise specified)	Expert review	Telephone interviews obtained by a professional	NS	NS	90%	72%

Lazarov, D.[39]	Pesticides (not otherwise specified)	3 levels probability by expert review	Questionnaire collecting occupational history, coded blindly according the Classification of the Italian Institute of Statistic and interpreted by a professional	1) not exposed; 2) 50-74% chance that exposure occurred; 3) >75%	<10 years (25 cases/ controls<br>11-20 (10/22)<br>21-30 (7/27)<br>>31 (27/37)	89%	100%
Adegoke, O.J.[40]	Pesticides (not otherwise specified)	JEM: Mustafa Dosemeci's NCI for Shanghai	Interviews by trained interviewers using standardized Chinese questionnaires; self-reported information combined with job-exposure matrix assessment.<br>Based on jobs held 3 years or more.	Duration of exposure: never, ever, < 10 years, ≥ 10 years<br>+ JEM (probability of exposure none, low, medium, high)	NS	91.4%	94%
Terry, P.D.[41]	Pesticides, insecticides and/or rodenticides	NS	Self- and proxy-interviews allowing classification of individuals as having been employed or not in an occupational category potentially associated with leukemia risk	Duration of employment at occupation: ever, never, ≥ 1 year, and duration of participation in hobby: never, ever, up to five years, 5+ years	NS	84%	66%
Kaufman, D.W.[42]	Gramoxone, huama herbicide; carbaryl; methyl parathion, glyphosate; trichlorofon, methylamidophos, paris green, dimethoate, dichlorvos/propoxur/cyfluthrin, DDT, spark, unspecified pesticide, unspecified herbicide, unspecified rodenticide	NS	Interview conducted by trained professional	Details were described (1) median of duration of use (2) median lifetime pesticide days (3) non occupational pesticide (4) pesticide used near home	NS	100%	100%

Wong, O.[43]	Pesticides, herbicides and fertilizers	Committee of local experts	Interview by trained professional	Dichotomous classification of “ever/never”	NS	97%	100%
Strom, S.S.[44]	Pesticides, herbicides and fertilizers	JEM: NCI	Interview and proxy-interview	The Job-Exposure Matrix was used to determine an intensity level of exposure: none, low, moderate, and high.	NS	87%	77%
Parodi, S.[45]	Pesticides (not otherwise specified)	NS	Interview through a standardized questionnaire by trained interviewers to obtain detailed information	Exposed, not exposed	NS	88%	81%
Poynter, J.N.[46]	List of specific insecticides, herbicides, fungicides and fumigants.	NS	A self-administered questionnaire	Duration of exposure 1) <1-10 years 2) >10 years	NS	58%	64%

JEM: job-exposure matrix; OPE: occupational pesticides exposure; NCI: National Cancer Institute (Dosemeci *et al. Epidemiology*. **5**, 124-127 (1994)); ALOHA: job-exposure matrix for chronic obstructive pulmonary diseases (Sunyer, *et al. Am J Respir Crit Care Med.* **157**, 512-517 (1998)); NS: not specified.

Table S3. Details on acute myeloid leukemia (AML) patients and control sources.

First author	AML source		Controls source	
Flodin, U.[33]	Multicentric	Patients from the hospitals of Linkoping, Norrkoping, Obrero and Umea and the cytological department of the hospital of Jonkoping in Sweden	Population	Two series of referents were used (1) controls were identified through a general population register matched for gender, age and dwellings (2) randomly from the same general population register of the catchment areas of the hospital, ages and the number of individuals totaled
Richardson, S.[34]	Multicentric	Patients from the clinical department of hematology at Hotel-Dieu, Paris, or in the department of hematology of Hospital Henri-Mondor in Creteil, near Paris.	Hospital	Controls were identified among hospitalized patients in other departments of the same hospitals
Crane, M.M.[35]	Multicentric	Patients from Texas Medical Centers: Methodist, St. Luke’s, Ben Taub, M.D. Anderson Cancer Center	Hospital	Located from admission printouts of participating hospitals
Ciccone, G.[36]	Monocentric	Patients from main hospital of Torino	Hospital and population	Two groups of controls were recruited (1) at hospital with no hematological or cancer diseases (including diseases of the digestive system, endocrine conditions and hypertension and CHD) (2) a random sample of the population living in the city of Torino
Mele, A.[37]	Multicentric	Patients from hospitals in Rome, Bologna and Pavia	Hospital	Controls were identified among out-patients seen in the same hematology departments, without a hematologic disorder, and among those with no cancer disorders seen in the same hospitals
Albin, M.[38]	Multicentric	Patients from Lund and Helsingborg	Population	Statistics Sweden selected 3 referents for each case. One referent in each matched set was randomly selected to be interviewed. When referents or their next-of kin could not be interviewed, other referents from the matched set were selected
Lazarov, D.[39]	Multicentric	Patients from two north west London Hospitals and the Haematology Department in Novi Sad, Yugoslavia	Hospital	Controls were identified among in-patients in the hospital from which the cases were recruited
Adegoke, O.J.[40]	Multicentric	Shanghai Cancer Registry which is composed of residents of urban Shanghai diagnosed with leukemia	Population	Controls were randomly identified from the general urban Shanghai population
Terry, P.D.[41]	Multicentric	Cancer and Leukemia Group B (CALGB), a multi-institutional cooperative cancer treatment group in the USA	Population	Controls were identified through a two-stage random digit dialing procedure

Kaufman, D.W.[42]	Monocentric	Patients from Siriraj Hospital (Bangkok)	Hospital	Controls were identified at Siriraj Hospital with diagnoses considered generally unrelated to the exposures of interest
Wong, O.[43]	Multicentric	Patients from 29 participating hospitals in Shanghai	Hospital	Controls were randomly identified from patients admitted to the same hospital
Strom, S.S.[44]	Monocentric	Patients from University of Texas M. D. Anderson Cancer Center	Population	Control were randomly identified without previous history of cancer in Texas population
Parodi, S.[45]	Multicentric	Patients from 11 areas in Italy: namely, provinces of Varese, Forlì, Siena, Latina, Ragusa, Imperia, Florence, Novara, Vercelli, and Verona plus the town of Turin	Population	Controls were identified by random sampling of the resident population
Poynter, J.N.[46]	Multicentric	Patients from Minnesota Cancer Surveillance System	Population	Controls were identified through the Minnesota State driver's license/identification card list

Table S4. Detailed Newcastle-Ottawa scale evaluation for studies included in the meta-analysis.

First author and reference	Selection of cases and controls				Comparability	Exposure assessment			Total
	Cases definition	Cases representativeness	Control selection	Control definition	Matched for age and gender	Ascertainment of exposure	Same method for cases and controls	Non-response rate	
Flodin, U.[33]	0	0	1	0	2	0	1	0	4
Richardson, S.[34]	1	1	0	1	2	1	1	1	8
Crane, M.M.[35]	1	1	0	1	2	0	1	1	7
Ciccone, G.[36]	1	1	0	0	2	0	1	1	6
Mele, A.[37]	1	1	0	1	0	0	1	0	4
Albin, M.[38]	1	1	1	0	2	0	1	0	6
Lazarov, D.[39]	1	1	0	1	2	0	1	1	6
Adegoke, O.J.[40]	0	1	1	0	2	1	1	0	6
Terry, P.D.[41]	1	1	1	0	2	0	1	0	6
Kaufman, D.W.[42]	1	1	0	1	2	0	1	1	7
Wong, O.[43]	1	1	0	1	2	1	1	1	8
Strom, S.S.[44]	1	1	1	1	2	0	1	1	8
Parodi, S.[45]	1	1	1	0	2	0	1	1	7
Poynter, J.N.[46]	1	1	1	1	1	0	1	0	6

Table S5. Reported funding of included studies.

First author and reference	Funding
Flodin, U.[33]	Swedish Work Environment Funding
Richardson, S.[34]	Inserm – Institut National de la Santé et de la Recherche Médicale
Crane, M.M.[35]	NIH K07
Ciccone, G.[36]	EEC-Europe Against Cancer Programme, Associazione Italiana per le Ricerche sul Cancro
Mele, A.[37]	Italian Ministry of Health, grant 500.4/RSC/70.18/T/1886, and by the Consiglio Nazionale della Ricerche Bilateral Project Italia-USA, grants 87.00186.04, 88.00600.04, and 89.02995.04.
Albin, M.[38]	Swedish Council for Work Life Research, Swedish Cancer Society, Medical Faculty of Lund University, Lund University Hospital, Gunnar, Arvid and Elisabeth Nilssons Research Foundation, and PREEM Research Foundation.
Lazarov, D.[39]	NR
Adegoke, O.J.[40]	Young Scientist Foundation, Chinese Ministry of Public Health; Grant (1U54-CA9140801) from the National Cancer Institute and National Institutes of Health.
Terry, P.D.[41]	NR
Kaufman, D.W.[42]	Thailand Research Fund, Commission on Higher Education.
Wong, O.[43]	Benzene Health Effect Consortium, American Petroleum Institute
Strom, S.S.[44]	NCI, National Institute of Environmental Health Sciences
Parodi, S.[45]	NCI grant, Europe Against Cancer Programme, Lega Italiana per la Lotta contro I Tumori
Poynter, J.N.[46]	NCI R01

NR: not reported

Table S6. Variables included in the final model from the seven studies reporting adjusted odds ratios.

First author and reference	Age*	Gender*	Prior radiation or chemotherapy exposure*	Benzene and other solvent exposure*	Ethnicity	Geographical zones	Smoking	Education	Income	Other
Richardson, S.[34]	X	X	X		X	X [a]				
Ciccone, G.[36]	X					X [a,b]	X			
Mele, A.[37]	X	X				X [a]		X		occupations
Adegoke, O.J.[40]	X	X							X	
Terry, P.D.[41]	X	X			X	X [c]	X	X		proxy respondent
Kaufman, D.W.[42]	X	X		X		X [a, d]			X	use of cellphones, occupational and non-occupational pesticide exposure, working with powerlines
Poynter, J.N.[46]	X	X	X			X [a, f]	X		X	

* refer to well-known risk factors of acute myeloid leukemia (Deschler, B. and Lübbert, M. *Cancer,* **107**, 2099-2107 (2006))

[a] of residency, [b] of birth, [c] not detailed, [d] living near powerlines, pesticides used near the home, [f] living near a farm

Table S7. Sensitivity analysis including results from the study of Bassig *et al*.

Pesticide/metabolite		Meta-analysis OR	Lower 95%CI	Upper 95%CI	p-value
**Tertile 2 versus tertile 1**	Oxychlordane	1.51	1.10	2.07	0.01
	trans-Nonachlor	1.51	1.10	2.07	0.01
	Heptachlor epoxide	1.50	1.09	2.06	0.01
	p,p'-DDT	1.51	1.10	2.07	0.01
	p,p'-DDE	1.49	1.09	2.05	0.01
	o,p'-DDT	1.47	1.08	2.01	0.01
	Mirex	1.51	1.10	2.08	0.01
	Hexachlorobenzene	1.44	1.05	1.97	0.02
	Dieldrin	1.51	1.10	2.07	0.01
	g-Hexachlorocyclohexane	1.50	1.09	2.06	0.01
	b-Hexachlorocyclohexane	1.48	1.08	2.03	0.01
**Tertile 3 versus tertile 1**	Oxychlordane	1.51	1.10	2.08	0.01
	trans-Nonachlor	1.51	1.10	2.07	0.01
	Heptachlor epoxide	1.51	1.10	2.08	0.01
	p,p'-DDT	1.51	1.10	2.08	0.01
	p,p'-DDE	1.51	1.10	2.07	0.01
	o,p'-DDT	1.50	1.09	2.06	0.01
	Mirex	1.50	1.09	2.06	0.01
	Hexachlorobenzene	1.49	1.09	2.05	0.01
	Dieldrin	1.51	1.10	2.08	0.01
	g-Hexachlorocyclohexane	1.47	1.07	2.02	0.01
	b-Hexachlorocyclohexane	1.50	1.09	2.06	0.01

Table S8. Stratified pooled odds ratio of the relationship between pesticide exposure and risk of acute myeloid leukemia.

Analyzed strata		No. studies	No. AML	No. controls	Reference number	Pooled OR [95%CI]	Heterogeneity tests:	Publication biais Egger's test p-value
							Q-test p-value (I^2)†	
Studies design	**AML population**							
	Multicentric	11	3,18	8,31	32, 33, 34, 34, 37, 38, 39, 40, 41, 43, 45	1.16 [0.93-1.44]	0.10 (38%)	0.55
	Monocentric	3	775	1,638	36, 42, 44	4.25 [2.97-6.09]	0.47 (0%)	0.84
	**Control population[a]**							
	Population-based	7	2,532	5,572	37, 38, 39, 40, 41, 43, 45	1.34 [0.76-2.34]	<0.001 (85%)	0.82
	Hospital-based	6	1,373	4,130	32, 33, 34, 35, 42, 44	1.55 [1.17-1.98]	0.29 (20%)	0.62
	**Study quality**							
	NOS≤6	7	1,973	4,569	36, 37, 40, 41, 43, 44, 45	1.20 [0.82-1.74]	0.04 (51%)	0.25
	NOS>6	7	1,982	5,379	32, 33, 34, 35, 38, 39, 42	1.80 [0.17-2.78]	<0.001 (80%)	0.75
	**Risk of bias[b]**							
	Unclear or high	4	1,713	4,749	35, 36, 39, 41, 42, 43, 44, 45	1.24 [0.85-1.82]	0.08 (45%)	0.51
	Low	6	2,242	5,199	32, 33, 34, 37, 38, 40	1.79 [1.07-2.97]	<0.001 (86%)	0.86
Pesticide exposure recording methods	**Exposure assessment**							
	Other	2	464	1,702	40, 45	0.92 [0.67-1.26]	0.56 (0%)	NA
	PTP	12	3,491	8,246	32, 33, 34, 35, 36, 37, 38, 39, 41, 42, 43, 44	1.64 [1.15-2.33]	<0.001 (75%)	0.76
	**Job exposure matrix or expert review**							
	No	6	1,625	6,000	32, 39, 40, 43, 44, 45	1.12 [0.84-1.51]	0.16 (37%)	0.15
	Yes	8	2,330	3,948	33, 34, 35, 36, 37, 38, 41, 42	1.82 [1.12-2.95]	<0.001 (76%)	0.55
Cofounding factors analysis	**Adjusted OR**							
	Yes	7	2,172	4,815	33, 35, 38, 39, 41, 42, 45	1.51 [0.95-2.40]	< 0.001 (81%)	0.54
	No	7	1,783	5,133	32, 34, 36, 37, 40, 43, 44	1.52 [0.93-2.48]	0.004 (67%)	0.32
Studied populations	**Geographical zone**							
	Europe	7	1,208	4,595	34, 36, 39, 41, 42, 44, 45	1.25 [0.87-1.82]	0.04 (53%)	0.26
	North America	4	1,702	2,651	35, 38, 40, 43	1.72 [0.55-5.43]	<0.001 (91%)	0.75
	Asia	3	1,045	2,702	32, 33, 37	1.74 [1.32-2.30]	0.33 (9%)	0.81
	**Inclusion periods**							
	Before 1995	10	2,103	5,764	34, 35, 36, 39, 37, 41, 42, 43, 44, 45	1.23 [0.91-1.66]	0.16 (31%)	0.53
	After 1995	4	1,852	4,184	32, 33, 38, 40	2.03 [1.07-3.85]	<0.001 (92%)	0.41
	**AML**							
	Other[c]	9	2,483	7,507	33, 34, 35, 37, 39, 40, 41, 44, 45	1.13 [0.89-1.45]	0.05 (50%)	0.46
	De novo	5	1,472	2,441	32, 36, 38, 42, 43	2.81 [1.58-5.00]	0.03 (62%)	0.18
Pesticide subtypes[d]	**Herbicides**	4	1,379	3,953	33, 40, 44	1.18 [0.89-1.57]	0.25 (26%)	0.41
	**Insecticides**	3	876	1,957	33, 34	1.47 [1.15-1.88]	0.92 (0%)	NA
	**Fumigants**	2	1,127	2,792	33, 40	1.52 [0.86-2.67]	0.82 (0%)	NA
**All studies**		**14**	**3,955**	**9,948**	**All included [33-46]**	**1.51 [1.10-2.08]**	**<0.001 (76%)**	**0.82**

Subgroups are in ascending order ordered according to their mean effects. [a] Cicconne *et al.*[42] not included: hospital- and population-based; [b] risk of bias classification was performed using correspondence analysis described in Fig S3 [c] Other: de novo status was not mentioned or 0.69% of secondary AML in Wong *et al.*[19]; [d] Only studies reporting specific pesticide exposure were included for this subset analysis. NOS: Newcastle-Ottawa scale; PTP: peer-to-peer interviews.

Fig S1. Inclusion periods and date of publication of studies included in the meta-analysis.

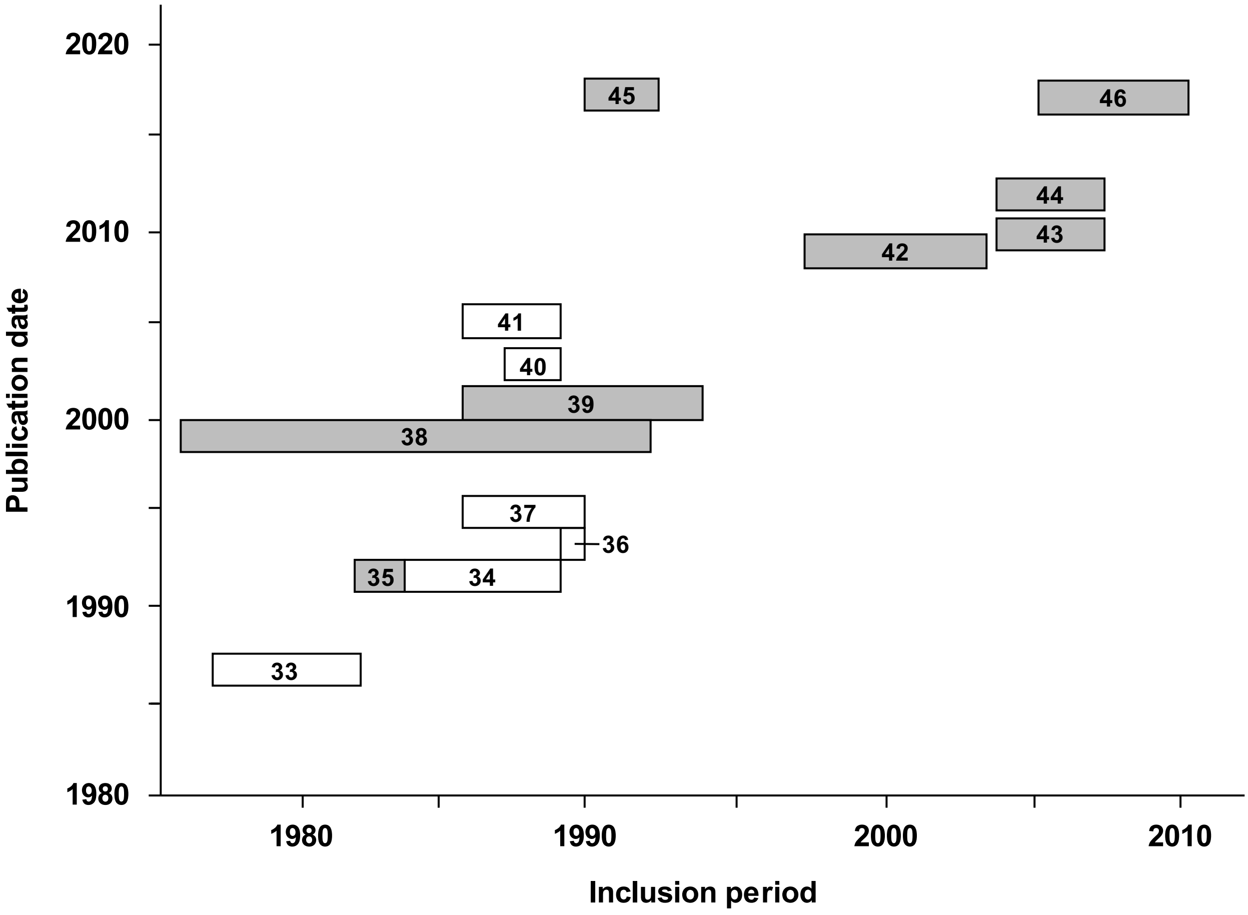

Studies are identified by their position in the reference list. White rectangles represent references included in the previous meta-analysis published in 2007[18].

Fig S2. Graphical representation of inclusion age heterogeneity between included studies.

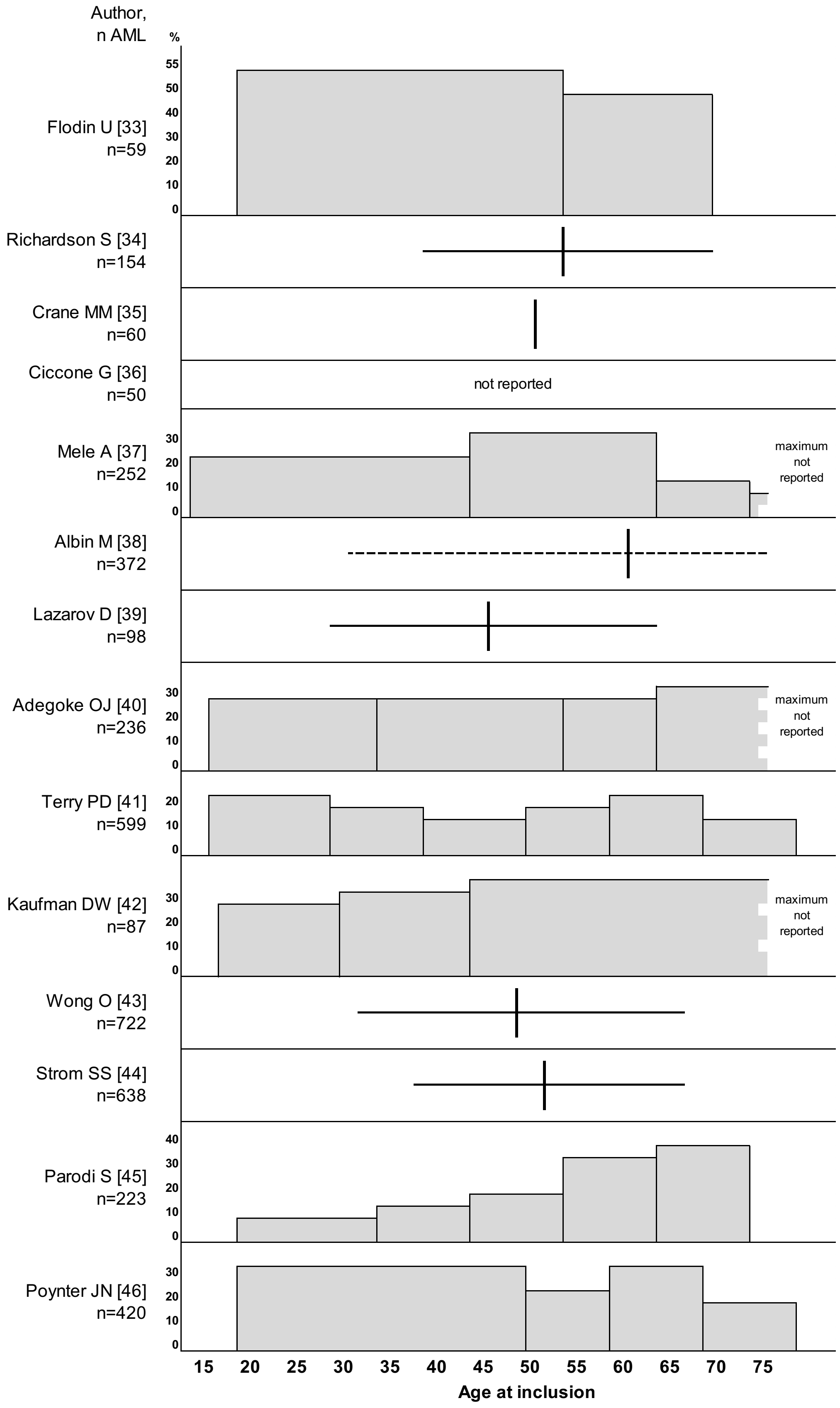

Bar plots represent the proportions of patients included. Vertical lines represent the mean and horizontal lines the standard deviation except for Albin 2000 where vertical and horizontal lines represent the median and 10-90% deciles.

Fig S3. Bias assessment within each study.

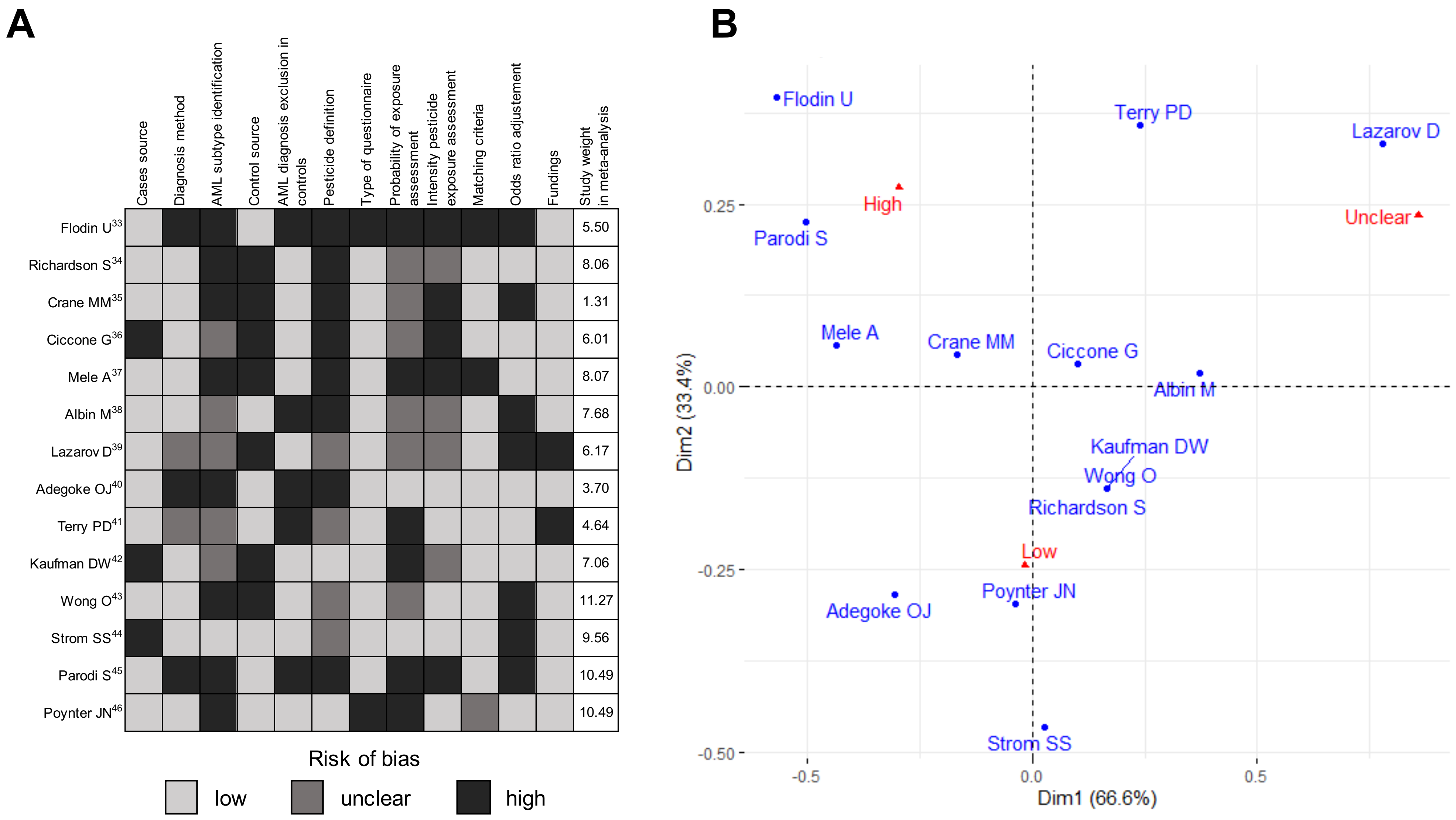

(A) Each study was assessed with three levels of risk of bias (low, unclear and high, Table S1). The weight of studies in the overall meta-analysis is represented. (B) Using the distribution of low, unclear and high risk (red) among each study included (blue), a correspondence analysis was performed to identify groups of studies according to the risk of bias.

Fig S4. Outlier studies identification with Q-Q plot.

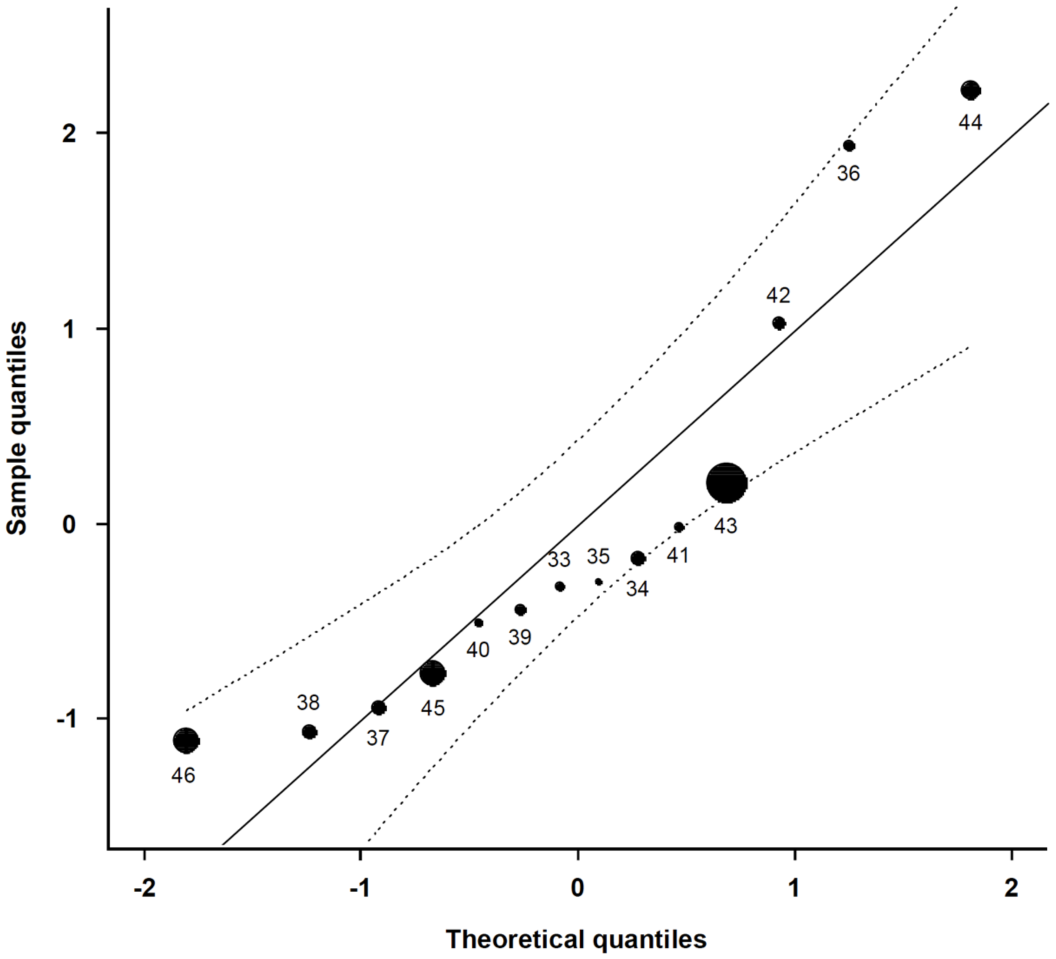

Studies are identified by their position in the reference list. The plot size is proportional to the weight of each study is the pooled odds ratios.

Fig S5. Frequency of acute myeloid leukemia over time together with pesticide usage in the USA.

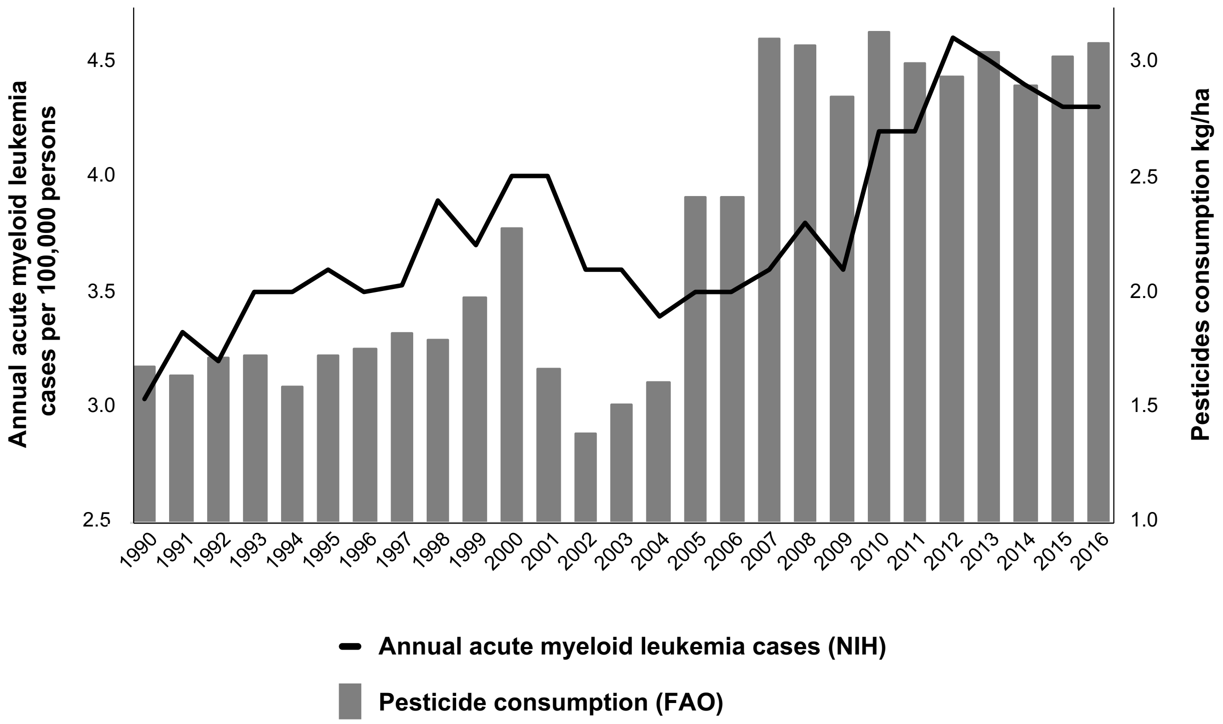

Pesticide use in kg/ha in the USA since 1990 according to the Food and Agriculture Organization (FAO) New cases of AML per 100,000 persons according to the national institute of health (NIH).

We compared available USA data from the FAO and National Institute of Health (NIH) on the annual incidence of AML and concomitant consumption of pesticides since 1990. This disclosed a weak positive correlation between these two variables through linear regression analysis (R^2=0.42).

NIH data available at:

https://seer.cancer.gov/csr/1975_2016/browse_csr.php?sectionSEL=13&pageSEL=sect_13_table.08

FAO data available at:

http://www.fao.org/faostat/en/#data/EP/visualize
